# Rethinking Fragility Fractures in Type 2 Diabetes: The Link between Hyperinsulinaemia and Osteofragilitas

**DOI:** 10.3390/biomedicines9091165

**Published:** 2021-09-06

**Authors:** Isabella D. Cooper, Kenneth H. Brookler, Catherine A. P. Crofts

**Affiliations:** 1 Translational Physiology Research Group, School of Life Sciences, University of Westminster, 115 New Cavendish Street, London W1W 6UW, UK; 2 Research Collaborator, Aerospace Medicine and Vestibular Research Laboratory, Mayo Clinic, Scottsdale, AZ 85259, USA; brookler.kenneth@mayo.edu; 3 School of Public Health and Interdisciplinary Studies, Faculty of Health and Environmental Sciences, Auckland University of Technology, Auckland 0627, New Zealand; catherine.crofts@aut.ac.nz

**Keywords:** hyperinsulinaemia, beta hydroxybutyrate, osteoporosis, type 2 diabetes, fragility fractures, bone mineral density, osteocalin, vitamin D, collagen, hydroxyapatite

## Abstract

Patients with type 2 diabetes mellitus (T2DM) and/or cardiovascular disease (CVD), conditions of hyperinsulinaemia, have lower levels of osteocalcin and bone remodelling, and increased rates of fragility fractures. Unlike osteoporosis with lower bone mineral density (BMD), T2DM bone fragility “hyperinsulinaemia-osteofragilitas” phenotype presents with normal to increased BMD. Hyperinsulinaemia and insulin resistance positively associate with increased BMD and fragility fractures. Hyperinsulinaemia enforces glucose fuelling, which decreases NAD+-dependent antioxidant activity. This increases reactive oxygen species and mitochondrial fission, and decreases oxidative phosphorylation high-energy production capacity, required for osteoblasto/cytogenesis. Osteocytes directly mineralise and resorb bone, and inhibit mineralisation of their lacunocanalicular space via pyrophosphate. Hyperinsulinaemia decreases vitamin D availability via adipocyte sequestration, reducing dendrite connectivity, and compromising osteocyte viability. Decreased bone remodelling and micropetrosis ensues. Trapped/entombed magnesium within micropetrosis fossilisation spaces propagates magnesium deficiency (MgD), potentiating hyperinsulinaemia and decreases vitamin D transport. Vitamin D deficiency reduces osteocalcin synthesis and favours osteocyte apoptosis. Carbohydrate restriction/fasting/ketosis increases beta-oxidation, ketolysis, NAD+-dependent antioxidant activity, osteocyte viability and osteocalcin, and decreases excess insulin exposure. Osteocalcin is required for hydroxyapatite alignment, conferring bone structural integrity, decreasing fracture risk and improving metabolic/endocrine homeodynamics. Patients presenting with fracture and normal BMD should be investigated for T2DM and hyperinsulinaemia.

## 1. Introduction

In 2010, 3.5 million fragility fractures were sustained in 27 European Union countries, with an estimated economic burden cost of €37 billion. It is predicted that by 2025, the economic burden will continue to increase by 25% [1]. Further, in the largest five European Union countries plus Sweden (EU6), fragility fractures are predicted to increase from 2.7 million in 2017 to 3.3 million by 2030, with a €37.5 billion annual fracture-related cost in 2017, expected to increase by 27% in 2030 [2].

Increased fragility fractures are well documented in patients with type 2 diabetes mellitus (T2DM), a condition of chronic hyperinsulinaemia [3,4,5,6,7]. Decreased skeletal bone mineral density (L-BMD) is the phenotype of “classical” osteoporosis [8,9]. A higher BMD is considered to confer greater bone strength (fracture resistance). However, the T2DM bone fragility phenotype more often presents with normal to increased BMD (H-BMD) [7,9,10,11,12,13,14], and positively tracks with increased fracture risk [4,5,7,12,15]. In a cross-sectional non-intervention study of 146 Caucasian non-diabetic postmenopausal women with a mean age of 60 ± 2.7 years, HOMA-IR was found to be positively associated with volumetric bone mineral density (vBMD) [11]. In addition, increased insulin resistance (IR) and treatment with exogenous insulin therapy is positively associated with higher BMD and increased fragility fractures. A study following 5994 T2DM males ≥ 65 years of age, found a higher non-vertebral fracture risk in those using insulin (HR 1.74, 95% CI 1.13, 2.69) who also had a higher BMD [15]. Furthermore, in a population-based matched cohort study investigating primary care records of 2979 insulin users and 14,895 non-users, T2DM patients exposed to insulin therapy to manage glycaemia were found to have a 38% excess fracture risk [12]. In both H-BMD-associated T2DM “osteofragilitas” (bone fragility) and L-BMD osteoporosis phenotypes, there is an increase in bone fragility and a loss in tensile and/or torsion strength, and bone ductility, resulting in higher rates of fractures.

Hyperinsulinaemia drives the pathogenesis of T2DM, which may precede hyperglycaemia by up to 24 years [6,16]. Hyperinsulinaemia decreases osteoblastogenesis and propagates poorer-quality collagen production, a problem further compounded by hyperglycaemia increasing glycation damage on new or existing bone collagen. Hyperinsulinaemia drives chronic osteocyte distress via excess ceramide synthesis, which increases cellular reactive oxygen species (ROS) [17,18,19,20], leading to a unique type of mineralisation within their lacunae, coined by Bell, Kayser and Jones as “living fossilisation” [21,22,23,24,25]. Concomitantly, hyperinsulinaemia decreases osteoclastogenesis, thus impeding the bone resorption needed to enable homeodynamic bone remodelling—a marker of good health [7,26] —and this results in a form of hyperinsulinaemia-hyperglycaemia pseudo-osteopetrosis. Combined, these hyperinsulinaemia-driven effects result in the increased bone mineral density seen in people with T2DM.

Osteocytes are the backbone of bone health, and consequently major players in whole-body metabolism [27]. Osteocytes are able to directly mineralise and resorb bone and are central mediators in the regulatory control of osteoblasts and osteoclasts [28,29,30,31,32,33,34,35,36]. Chronic hyperinsulinaemia diminishes the replenishment of osteocytes and drives the living fossilisation of the ones in existence, leading to the loss of the osteocyte’s dynamic orchestration of bone remodelling [21,22,23,24,25,37]. We propose chronic hyperinsulinaemia provides a plausible explanation, a unifying theory of the mechanisms of action, for the increased BMD and bone fragility “osteofragilitas”, that leads to the increased fracture rates seen in the T2DM bone phenotype.

## 2. Osteocytes: Mediators of Bone Remodelling and Metabolic Heath

Within the adult skeleton, osteocytes comprise 90% to 95% of the total bone cells [37]. Osteocytes are terminally differentiated cells from the osteoblast lineage. They are the heavyweight lifters in the living dynamic bone tissue that not only provides a physical scaffold for the body, but is also fundamental in endocrine regulation and whole-body metabolism [14,38,39]. Osteocytes embed within the bone after collagen formation by osteoblasts, of which some of these osteoblasts are fated to differentiate into the embedding osteocytes [37]. The osteocytes, along with their neighbouring osteoblasts, continue to form mineralised bone onto the collagen scaffold, in the process forming the hydroxyapatite lacuna chamber around the embedding osteocytes [27]. Continual morphological changes take place in the process of osteocytogenesis, leading to cells that bear little resemblance in structure to their predecessors, appearing visually more like neurons. Osteocytes have on average 50, and up to 100 dendrites, extending through highly connected intricate tunnels, called the canaliculi. The canaliculi are formed and maintained by the occupying osteocytes [40], and enable physical connection to other osteocytes and to the outside surface of bone, to osteoblasts, osteoclasts and the vasculature [41].

The lacunocanalicular system is a fluid-filled space, separated from the mineralised component of bone and maintained by the resident osteocytes [22]. The osteocytes are the workhorse physical mechano-sensors and mediators of bone remodelling, sensing and responding to mechanical stress via their strategic distribution and network of a vast number of connected dendritic processes that enable intercellular communication. Their network forms the essential antenna to detect fluid shear stress, which enables the translation of mechanical stimuli into biochemical messages. Osteocytes, in turn, feed these signals forward via autocrine and paracrine mechanisms, to elicit homeostatic adaptive responses, from regulating bone remodelling in order to provide sensitive and continual changes to whole-body mineral needs, to effecting distant organ responses via endocrine signalling [22,41].

## 3. Dendritic Connectivity Is Essential for Function and Viability

Osteocytes connect to one another via their dendritic processes which form the osteocyte-lacunocanalicular network, and their health and viability are dictated by their dendritic connectivity [41,42]. A compromise in osteocyte health diminishes their ability to actively inhibit mineralisation of their pericellular space [25,28], consequently reducing their connectivity. This further compromises the health of deeper osteocytes that become cut off from receiving nutrients which are no longer able to be delivered through fluid movement via the canalicular tunnels. Furthermore, osteocyte connectivity is required for the transduction of load-induced fluid flow that enables their mechanical sensory system to decrease apoptosis and increase osteocytogenesis [43]. Maintenance of dendritic connectivity allows osteocyte-directed regulation of osteoblasts and osteoclasts, in addition to their own capacity to directly resorb bone [29,30,34,35,36].

**Figure 1:**
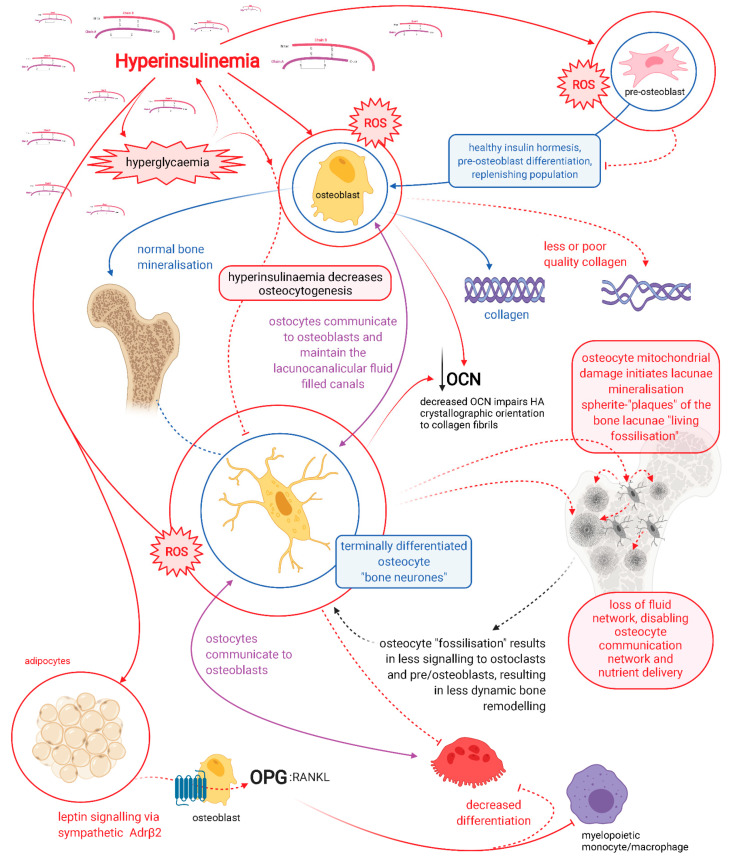
Schematic representation showing the dynamic role of osteocytes in the regulation of healthy and dysregulated bone. Beta-adrenergic receptor (Adrβ2), hydroxyapatite (HA), osteocalcin (OCN), osteoprotegerin (OPG), receptor activator of nuclear factor kappa-β ligand (RANKL), and reactive oxygen species (ROS). Red lines indicate hyperinsulinaemia-driven pathology pathways. Healthy physiology indicated with blue and purple lines.

It is highly likely that osteocytes rather than osteoclasts are responsible for bone resorption in the basal condition, as a function of whole-body mineral homeostasis, and that osteoclast bone resorption serves to function in the acute action/need/stress response [34]. Osteocytes from lactating mice have been shown to express markers thought to be specific to osteoclasts. Their osteocyte lacunae were found to be enlarged, suggesting localised bone resorption, providing evidence of osteocytic-osteolysis [44]. Patients with T2DM have decreased levels of carboxy-terminal collagen crosslinks (CTX) bone resorption marker, indicating a lower bone turnover [13]. Osteocytes regulate both osteoblast and osteoclast differentiation and function, and are thus master regulators of dynamic bone remodelling, a function of healthy physiology (Figure 1) [45].

## 4. Hyperglycaemia Increases Advanced Glycation End-Product Formation in Bone Collagen

The anabolic hormone insulin is required at the basal level for healthy bone formation [3]. However, chronic hyperinsulinaemia surpasses this threshold in a dose and duration manner. When coupled with hyperglycaemia, it results in the production of poorer-quality, more rigid collagen and glycation damage of existing collagen [46,47]. Together, they drive increasing BMD by promoting skeletal mineral acquisition that is fragile in structure [22,48]. Hyperglycaemia and hyperinsulinaemia increase advanced glycation end-product (AGE) formation. Increased glycation on fibrillar collagen negatively affects bone quality [14]. In human tissue, the most abundant AGE is glucosepane, a lysine–arginine cross-linking, that forms the major AGE in bone type 1 collagen. Hyperglycaemia is one of the leading causes of AGE formation, affecting the structural and biochemical properties of protein binding sites, rendering them unrecognisable to other proteins and enzymes [47].

Hyperglycaemia-driven AGE formation of glucosepane in bone collagen causes a decrease in viscoelasticity and increases the production of a stiffer collagen, resulting in negative effects on the mechanical properties of load-bearing collagen in bone. This causes bone toughness to decrease, while a greater accumulation of AGE in bone results in increased fracture risk [47]. Furthermore, an increase in stiffer/rigid collagen production occurs due to increased glycation effects on the vasculature, leading to increased hypoxia in the microenvironment [14]. Hypoxia then compromises the osteoblasts’ capacity to generate sufficient ATP for collagen synthesis and for differentiation into osteocytes (Figure 1). Both of which are very energy intensive processes, requiring an efficient mitochondrial capacity to generate ATP via oxidative phosphorylation (OxPhos) [49,50,51,52]. In addition, hyperinsulinaemia and hyperglycaemia inhibit beta-oxidation, whilst increasing mitochondrial (mt) reactive oxygen species (ROS) formation [53]. This leads to increased H_2_O_2_ production, causing damage to intracellular protein synthesis machinery and consequently synthesis of poorer quality collagen. The typical methodology of assessing bone quality is via dual x-ray absorptiometry, however, this method is unable to detect the collagen aspect of bone quality [14]. As a result, there is an increased frequency in missing early detection of hyperinsulinaemia-hyperglycaemia osteofragilitas fracture risk, which typically does not present with L-BMD. This suggests that BMD alone is a poor proxy/diagnostic marker for fracture risk in hyperinsulinaemic individuals.

**Figure 2:**
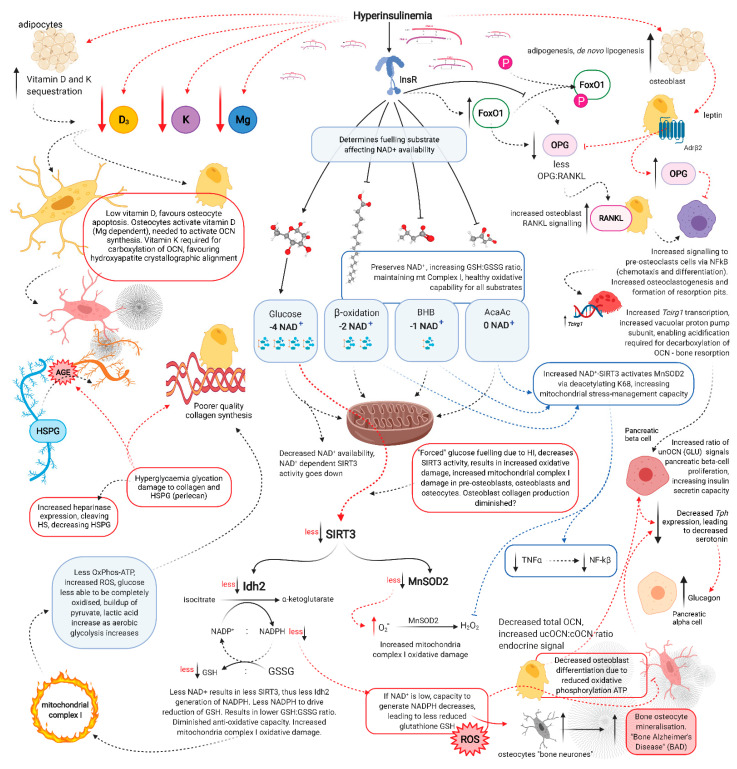
Schematic representation of hyperinsulinaemia effects on cellular oxidative state, and bone homeodynamics. Acetoacetate (AcAc), adenosine triphosphate (ATP), advanced glycation end-products (AGE), beta-adrenergic receptor 2 (AdrB2), beta-hydroxybutyrate (BHB), heparan sulphate (HS), heparan sulphate proteoglycan (HSPG), hydrogen peroxide (H_2_O_2_), hyperinsulinaemia (HI), insulin receptor (InsR), isocitrate dehydrogenase 2 (Idh2), forkhead box O1 (FoxO1), glutathione oxidised form (GSSG), glutathione reduced form (GSH), lysine 68 (K68), magnesium (Mg), manganese superoxide dismutase 2 (MnSOD2), nicotinamide adenine dinucleotide (NAD+), nicotinamide adenine dinucleotide phosphate (NADP+), nuclear factor-kB (NF-kB), osteocalcin carboxylated (Gla-OCN or cOCN), osteocalcin un(der)carboxylated (Glu-OCN or ucOCN), osteoprotegerin (OPG), oxidative phosphorylation (OxPhos), receptor activator of nuclear factor-kB ligand (RANKL), reactive oxygen species (ROS), sirtuin 3 (SIRT3), superoxide (O_2_^−^), tumour necrosis factor α (TNFα), T cell immune regulator 1 (*Tcirg1*), tryptophan hydroxylase (*Tph*), vitamin D (D_3_), and vitamin K (K).

Hyperinsulinaemia “enforces” cellular glucose substrate fuelling [54], and downregulates beta-oxidation by increasing intracellular ceramide production [20]. Excess ceramide production compromises mtOxPhos capacity, by increasing dynamin-related protein 1 (Drp1) synthesis. Drp1 functions to increase mitochondrial fission, in addition to increasing the production of mtROS such as: superoxide (O_2_^−^), hydroxyl radical (^−^OH) and hydrogen peroxide (H_2_O_2_) [17,20,55]. Concomitantly, ATP production from glucose oxidation reduces the intracellular pool of nicotinamide adenine dinucleotide (NAD+), consuming four NAD+ in the production of two acetyl moieties, in comparison to beta-oxidation, ketolysis or oxidation of acetoacetate, which consume two, one and zero respectively (Figure 2) [56]. Thus, ATP synthesis that is increasingly reliant on glucose oxidation, negatively impacts the availability of NAD+.

Sirtuin-3 (SIRT3) regulates the synthesis of endogenous antioxidant enzymes such as mitochondrial manganese superoxide dismutase (MnSOD2) and NADPH-dependent production of reduced glutathione (GSH) [56,57]. An increased reliance on glucose fuelling and its effect on NAD+ availability, leads to a reduction in SIRT3 activity, since SIRT3 is NAD+ dependent. This in turn decreases signals for the transcription and synthesis of MnSOD2 and GSH [56,57]. Furthermore, hyperinsulinaemia, diminishes the oxidative buffering capacity of the cellular redox antioxidant GSH [58]. Beta-oxidation activity dramatically increases in osteoblasts as they mature. If beta-oxidation is diminished and/or inhibited, metabolic demand cannot be met, resulting in a decrease in precursor cellular differentiation capability, as osteoblastogenesis and osteocytogenesis are energy intensive processes [49,51,52]. Bone neither stores nor synthesises any significant amount of fat. Thus, fatty acids delivered via chylomicron remnants (CR) and low-density lipoproteins (LDL) to bone are more likely to be used for ATP synthesis via beta-oxidation [51].

## 5. Hyperinsulinaemia Increases Osteocyte Mitochondrial Fission and Disassociation from the Endoplasmic Reticulum

Healthy osteocytes are able to transfer their functional mitochondria to neighbouring distressed osteocytes via their physically connected dendrites [42]. Mitochondrial transfer between osteocytes declines with age and with decreased dendritic connections [22,28,42,59]. Osteocytic rescue of their distressed neighbours via mitochondrial transfer is dependent on their mitochondria associating with the endoplasmic reticulum (ER) and on their dendrite connectivity, which requires the maintenance of their lacunocanalicular tunnels via inhibiting excessive mineralisation [21,29,31,42]. Hyperinsulinaemia increases mtROS production via ceramide synthesis which increases mitochondrial fission and ER stress [20,60]. This results in less mitochondrial fusion, which is necessary for beta-oxidation, and also leads to decreasing the osteocytes’ capability to transfer mitochondria to adjacent distressed osteocytes. This osteocytic mitochondria transfer process is dependent on mitochondrial association with the ER (ER-mito), driven by the protein guanosine triphosphatase mitofusin 2 (Mfn2), which localises to the outer mitochondrial membrane (OMM), enabling the tethering of the mitochondrion to the ER [42]. Reductions in Mfn2 expression leads to reductions in mitochondrial fusion and distribution, which further decreases the ability of healthier osteocytes to rescue their distressed neighbours [22]. Hyperinsulinaemia increases the Drp1:Mfn2 ratio. As a result this favours mitochondrial fission and ER-mito disassociation, leading to decreased OxPhos ATP synthesis and increased mtROS production [20,61]. Interestingly, osteocytes are under a form of social control, as they must receive signals from other cells for their survival, which is reliant on their connectivity [48]. A fall in the number of viable osteocytes or dendritic connections, below an essential minimum number (a threshold), or excessive lacunocanalicular mineralisation, may result in a severely compromised osteocyte-network signal transduction capacity, which in turn would impair dynamic bone remodelling [48]. Osteocytes actively maintain the lacunae-canalicular space, enabling fluid flow dynamics that ensures the provision of nutrients and signalling molecules such as growth factors and cytokines, in addition to facilitating the removal of waste. This is essential for the deeper embedded osteocytes and osteoblasts, to remain viable and function healthily [29]. Chronic hyperinsulinaemia may contribute to significant detrimental remodelling of dendrites and the lacunae-canalicular space, where the number (threshold) of osteocytes affected may result in a profoundly negative outcome.

## 6. MnSOD2 and SIRT3 Required for Osteoblastogenesis and Osteocytogenesis

Chronic hyperinsulinaemia and hyperglycaemia drives the pathogenesis of T2DM, chronic kidney disease (CKD) and atherosclerosis, conditions that are associated with increased rates of fractures independent of L-BMD. Hyperinsulinaemia with hyperglycaemia likely causes a decrease in osteocyte population and/or functional capacity leading to hyperinsulinaemia-*osteofragilitas* [4,38,39,62,63]. Glucose restriction increases osteoblast-to-osteocyte specification, and conversely hyperglycaemia reduces osteoblast gene expression of *Osx, Bglap* or *Dkk1*, leading to a reduction in osteocytogenesis [37]. Furthermore, hyperglycaemia decreases osteocyte connectivity and population. Viable osteocytes are needed to signal pre-osteoblastogenesis and osteocytogenesis. Therefore, hyperglycaemia’s negative impact on osteocyte numbers and dendritic connectivity, may result in a detrimental impact on the maintenance of the osteocyte-lacunocanalicular network. As a result, this would impair the osteocytes’ role in dynamic bone remodelling in the basal state. This is likely a contributing factor to the lowered state of bone remodelling seen in hyperinsulinaemic T2DM and CVD patients [45].

Hyperinsulinaemia/glycaemia mediated decreases in NAD+ availability, consequently decreases antioxidants MnSOD2 and SIRT3 activity, this leads to a negative impact on osteoblast differentiation [64]. Both MnSOD2 and SIRT3 are required to regulate mitochondrial stress, to enable cellular differentiation and bone formation [64]. MnSOD2 dismutes mitochondrial superoxide, thus protecting complexes I and II of the mitochondrial electron transport chain (ETC), where OxPhos occurs. Glucose restriction increases ketolysis, which increases the redox span between complex I and III [54,65]. This decreases electron leakage at these sites, which reduces the formation of superoxide. Glucose restriction concomitantly leads to an increase in NAD+ dependent SIRT3 activity, which increases deacetylation of MnSOD2 lysine residues: 53, 68 and 89 (K53, K68, K89), consuming NAD+ in the process [56,57,66,67,68,69]. This consequently upregulates MnSOD2 antioxidant activity, resulting in improved ROS management [70]. With improved ROS management and mt-OxPhos capacity, due to glucose restriction and ketolysis, this maintains osteocytogenesis, viability and connectivity. Maintenance of osteocyte viability knock-on effect results in sustained dynamic bone remodelling, as well as regulation of osteoblast and osteoclast differentiation and activity.

## 7. Glucose Restriction Increases Glutathione Activity and Improved Cell Viability

Glucose restriction decreases insulin signalling, thereby enabling increased beta-oxidation and ketolysis, preserving NAD+ availability to enable upregulated SIRT3 activity which increases NADPH production [71]. This drives increased glutathione reductase activity, which increases the ratio of reduced to oxidised glutathione (GSH:GSSG). Thus enhancing the intracellular antioxidant capacity to combat ROS damage (Figure 2) [72].

Calorie restriction decreases glucose and insulin levels, whilst increasing fatty acid oxidation and ketogenesis. This is the metabolic phenotype of fasting. Mice under lower levels of glucose and insulin show a marked increase in SIRT3 expression and activity, consequently preventing age-related hearing loss, a condition associated with ageing, increased hyperinsulinaemia, and osteofragilitas conditions [73]. In a mouse model, elevated insulin and high glucose compromised mitochondria in osteocytes, increasing both cytoplasmic and mitochondrial ROS, driving an inverse correlation between glutathione and mitochondrial ROS. Additionally, an increasing compromise in osteocyte mitochondrial-function strongly correlates with increased impairments in skeletal health [74].

## 8. Glucose Restriction Enables Osteocytogenesis; Hyperglycaemia Inhibits It

Hyperglycaemia inhibits osteoblast differentiation into osteocytes, and reduces the osteoblasts bone mineralisation capacity. In an in vitro model, IDG-SW3 cells differentiated into osteoblasts were cultured in 1, 5 or 25 mmol/L of glucose, using an alizarin red assay, it was shown that the osteoblasts cultured in the higher glucose concentration had a decreased ability to form calcium deposits [37]. Furthermore, osteocytes cultured in 25 mmol/L of glucose, showed a decrease in osteoblast-to-osteocyte transition, assessed using osteocytic *Dmp*-GFP gene expression reporter. A negative correlation was found between glucose availability and osteocytogenesis, where osteoblast-to-osteocyte transition increased as glucose concentration decreased, assessed via fluorescence microscopy/cell sorting of *Dmp*-GFP expression. In short, glucose restriction increased osteocyte gene expression [37]. These results indicate hyperglycaemia may disturb, if not diminish osteoblast and osteocyte replenishment potential. Hyperinsulinaemia and hyperglycaemia impair osteoblastogenesis, osteocytogenesis and osteoclastogenesis, resulting in decreased dynamic bone remodelling. Patients with T2DM and CVD show marked decrease in bone remodelling [7,51].

In a situation of hyperinsulinaemia which potentiates and consolidates hyperglycaemia, osteoblast bone formation may become compromised over time, while osteoblast-to-osteocyte transition is also diminished. In this scenario, one would expect less evidence of bone mineralisation. However, if in the same hyperinsulinaemic and hyperglycaemic state, the existing osteocytes are also producing elevated levels of mtROS, osteocyte death would potentially incur vacancies in their lacunocanalicular space, which would result in a state of heightened structural fragility. This may phenotypically appear as L-BMD osteoporosis. However, hypothetically in a hyperinsulinaemic state, the “bone neurone” sensory osteocyte makes a “margin-call” [25,75,76], to fossilize instead of leaving an empty and thus structurally fragile space, so as to ensure structural rigidity at the very least.

Alternatively, the rationale may be simpler, where the osteocyte fossilisation occurs in the hyperinsulinaemic state, so as to aid in mineral accretion during times recognised as “feasting and energy abundance”. A plausible explanation would be hyperinsulinaemia occurring in summer and autumn, when there is greater availability of high carbohydrate foods, leading to increased levels and duration of insulin signals to the cells that there is a food abundance period. Insulin mediates de novo adipogenesis, chronic insulin signals to adipocytes to increase storage of fuel in the form of lipids (de novo lipogenesis) and to bone cells to save minerals “for a rainy day”, in expectation of typically less food abundance in winter. A similar physiological adaptation example for increasing storage of fuel, minerals and vitamins, is during pregnancy, which has a natural state of mild hyperinsulinaemia, in-order to provide for post-partum lactation [35,77].

Glucose restriction would historically normally occur during wintertime, with concomitant lowered insulin levels, as a result of decreased food abundance, especially the farinaceous kind. A low glucose and low insulin environment would facilitate bone remodelling and improved resorption, as beta-hydroxybutyrate (BHB) inhibits mineralisation, while acetoacetate activates mineralisation. Bone resorption increases either via increased osteoclast activity, or higher levels of BHB which decreases the localised pH [78]. This releases undercarboxylated osteocalcin (OCN), which would negatively regulate BHB synthesis through its action on regulating basal insulin secretion [79,80]. This keeps ketones in the physiologically healthy range [81]. The low glucose and insulin state that facilitates dynamic bone remodelling, produces opened-up spaces for osteoblasts to then begin matrix synthesis and renewed bone mineralisation activity. This is followed by transition into a new generation of fresh osteocytes to maintain the newer sections of the lacuna-canalicular network. However, if a glucose restriction phase does not occur, over time the pre-osteoblast population may become compromised, whilst over-fossilisation simultaneously occurs. Subsequent chronic hyperinsulinaemia induces excess osteocytic ROS production to a degree that osteocytes are no longer able to actively inhibit mineralisation of their lacunocanalicular space, which is required to enable their survival, as well as those they are further connected with. Combined, these effects may drive increasing bone density, while making the bones both brittle and fragile.

## 9. Pyrophosphate and Sclerostin

Osteocytes produce pyrophosphate (ePPi), an inhibitor of mineralisation and carbonate solubiliser of bone mineral and matrix, thus regulating mineralisation and the circulatory systemic levels of calcium and phosphate [82]. Hyperinsulinaemia increases mtROS and cellular pathology, compromising osteocyte health, leading to mitochondrial mediated living fossilisation of osteocytes within their lacunae. Magnesium-dependent spherite-mediated osteocyte mineralisation provides evidence to support this, where higher levels of magnesium are found in these micropetrosis fossilisation spaces formerly inhabited by osteocytes [23]. This potentially traps/entombs magnesium within the living fossilised bone; subsequently contributing to magnesium deficiency (MgD). Furthermore, MgD has its own effects on potentiating hyperinsulinaemia [83,84,85]. Culminating in a vicious feedforward cycle.

While bisphosphonates are known to work at inhibiting osteoclast bone resorption, they likely also work as an ePPi analogue. By assisting in inhibiting excessive mineralisation of the osteocytes lacunae, helping to maintain fluid flow and osteocyte connectivity where struggling osteocytes may fail to do so, thus saving the day in “assisting” the osteocytes in their job to be able to regulate dynamic bone turnover, a marker of metabolic health [45,86].

A density threshold level of osteocytes is required to maintain homeostatic bone remodelling, from sensing damage in-order to initiate repair processes [48], to mineral storage during pregnancy or in preparation for a winter with decreased food availability. Greater lacunae mineralisation has been detected with advanced age and/or untreated osteoporosis. Increased lacunae calcium content coupled with poorer quality matrix, contributes to increased bone brittleness and subsequent fragility [22,46].

Osteocytes synthesise sclerostin, a 22.5 kDa protein, which reduces osteoblast differentiation, consequently downregulating bone metabolism. Although there is a clear relationship between hyperinsulinaemia and sclerostin levels, further research is required. Theoretically, a decreased osteocyte mass should predict lower sclerostin levels. However, elevated sclerostin levels have been found in patients with CVD and chronic kidney disease (CKD), two groups who typically have osteofragilitas. Furthermore, osteogenic differentiation of vascular smooth muscle cells (VSMC) in CVD and CKD patients have been detected, where there is an increase in expression of sclerostin in aortic valve tissue [87,88,89,90]. This means caution is required when assessing bone and metabolic health via sclerostin levels.

## 10. Osteocytes Produce Alkaline Phosphatase

Alkaline phosphatase (ALP) is an extracellular membrane bound ectometalloenzyme that catalyses the hydrolysis of inorganic pyrophosphate (PPi) to phosphate (Pi) at an alkaline pH. Phosphate forms part of calcium hydroxyapatite crystals, and an increase in phosphate promotes mineralisation. Gene mutation of tissue-nonspecific alkaline phosphatase (TNAP) results in hypophosphatasia under-mineralisation, demonstrating the integral role of ALP in bone mineralisation [91].

Nucleotide pyrophosphatase phosphodiesterase (NPP1) inhibits the action of ALP by increasing the concentration of calcification inhibitor pyrophosphate (ePPi) [70]. Insulin and the fed-state reduces NPP1 gene (*Enpp1*) expression. Interestingly, fasting has been shown to increase its expression. In short, fasting increases the concentration of ePPi via NPP1, leading to inhibition of excessive mineralisation of the osteocyte lacunocanalicular space, which results in maintaining osteocyte viability, dendritic connectivity and consequent dynamic bone remodelling capacity. Insulin action leads to decreased ePPi concentration, subsequently decreasing the osteocytes ability to inhibit bone mineralisation, thus increased mineralisation of the osteocytes lacunocanalicular space occurs. The fasted state provokes the opposite effect, inhibition of mineralisation, and potentially enhances physiological levels of bone resorption via increased beta-hydroxybutyrate (BHB) [92].

If autumn were a time for humans to accumulate stored energy in preparation for a fasting period during winter, where foods available during autumn increase insulin secretion, activating energy storage mechanisms, logically mineral storage would also be required and may also be facilitated via insulin’s action on inhibiting NPP1 production of ePPi. This may provide an evolutionary explanation, where seasonal hyperinsulinaemia propagates increased osteocyte-lacunae mineralisation during autumn, as an adaptive survival mechanism. This would then be followed by a winter of fasting, which would subsequently lead to increased NPP1 activity, relinquishing back into the system the stored minerals. Hyperinsulinaemia T2DM could be described as a metabolic phenotype reflecting a constantly “fed-state”, the ever-lasting autumn. Thus, providing an explanation of the normal to increased BMD observed in people with T2DM.

## 11. Osteocalcin

Patients with T2DM and insulin resistance have significantly lower levels of circulating osteocalcin (OCN) than healthy controls [71,93,94,95,96]. OCN is a non-collagenous protein, largely synthesized by osteoblasts and osteocytes that retain their expression of OCN [97]. Levels serve as a marker of osteoblast and osteocyte health. Serum OCN levels positively correlates with: dynamic bone remodelling, decreased insulin resistance (IR), and reduced T2DM and CVD risk [96,98,99,100]. Much published research show OCN increases insulin secretion, with conclusions stating decreased OCN levels would result in decreased insulin synthesis and secretion, and result in impaired glucose homeostasis. However, when assessed in humans, low levels of OCN tracks with hyperinsulinaemia [96,101]. Ergo providing evidence that production of high levels of insulin does not require high levels of OCN.

## 12. Osteocalcin Endocrine Effects

Interestingly, OCN significantly increases insulin-independent glucose uptake, and even more so in the presence of insulin. This leads to increased insulin sensitivity, through reducing the amount of insulin required to facilitate glucose uptake [98]. Additionally, OCN production increases the expression of mitochondrial uncoupling protein 1 (UCP1) in adipocytes, leading to increased thermogenesis and mitochondrial biogenesis, thus increasing glucose and fatty acid oxidation capacity. Furthermore, OCN increases adipocyte production of adiponectin. However, caution is required when interpreting experiments involving administering exogenous OCN in animal studies, in which these animals are fed diets that do not induce hyperinsulinaemia (obesogenic for that species), that would mimic the main human causal factor for T2DM. In the hyperinsulinaemic state, adiponectin receptors Adr1/2 are downregulated [102]. The signalling dynamics and results elucidated from animal studies in which exogenous OCN is provided to metabolically normal, or genetically induced OCN deficient and/or OCN receptor KO mice, would likely not be the same as what would occur in hyperinsulinaemic humans with low OCN. For example, healthy insulin levels and insulin sensitivity plus exogenous OC*n* = X, whilst hyperinsulinaemia and insulin resistance plus exogenous OC*n* = Y.

When wild-type mice (healthy) were administered exogenous OCN, their adipose tissues were observed to have upregulated peroxisome proliferator-activated receptor gamma coactivator 1-alpha (PGC1α) and adiponectin. Both adiponectin and PGC1α expression and activation, lead to increased beta-oxidation and correspond to improved metabolism, insulin sensitivity and glucose homeostasis [103]. Effectively, metabolically healthy mice respond in a physiologically normal way to the OCN. The elevated OCN in this context, is thus able to further effect (positive feedforward) mechanisms that consolidate better glucose/fatty acid oxidation and ROS management. The marker then becomes a maker of good health. OCN levels and the carboxylated-to-undercarboxylated ratio (cOCN:ucOCN or Gla:Glu) act as surrogate markers of osteoblast and osteocyte health, and consequently bone quality, which are then able to actively function in endocrine homeostasis and metabolic health.

The effect of OCN on adipocytes include: improved insulin independent glucose uptake, increased adiponectin synthesis and “energy wastage” through thermogenesis, and decreased inflammatory cytokine production, leading to increased skeletal and muscular insulin sensitivity [98]. Greater insulin sensitivity decreases the amount of insulin required to achieve glucose homeostasis. Individuals who maintain normo-insulin levels via restricting carbohydrate intake, would likely maintain healthier osteoblasts and osteocytes. This enables healthy endogenous OCN synthesis, as is seen in healthy controls. In the context of normo-insulin and insulin sensitivity plus OCN, the result is X. Which is OCN increasing adiponectin synthesis and signalling in a none hyperinsulinaemia and normoglycaemia contextual environment. Furthermore, a reduced requirement and secretion of insulin, decreases excessive mitochondrial ROS production and subsequent downstream cellular pathophysiological adaptations.

## 13. Carboxylation of Osteocalcin

Post translational modification alters OCN into one of 3 isoforms, which affects bioavailability and activity of the bone-derived hormone. γ-carboxylation of OCN occurs before secretion from osteoblasts and osteocytes. γ-glutamyl carboxylase (GGCX) enzyme activity is dependent on availability of its cofactor vitamin K (in the reduced state), in order to carboxylate OCN on glutamic acid residues: Glu17, 21 and 24. The carboxylated (cOCN or Gla-OCN) form, is the most abundant form in bone extracellular matrix as carboxylation increases OCN affinity for the mineral component of bone hydroxyapatite [79,98]. The undercarboxylated and uncarboxylated forms, (ucOCN or Glu-OCN) are considered the biologically active hormone isoforms. All three isoforms are present in the blood [39]. OCN concentration in human blood ranges between 10 to 40 ng/mL [39]. Interestingly, in vitro experiments by Hill et al. show OCN in both carboxylated and uncarboxylated forms are biologically active, both are able to modulate glucose uptake and increase insulin sensitivity. However, the ucOCN form was more effective [98].

## 14. Osteocalcin and Insulin

Much research has shown that insulin signalling induces osteoblasts and osteocytes to produce OCN and in vivo studies indicate that plasma OCN stimulates an increase in pancreatic beta cell differentiation/proliferation via increasing cyclin D1, D2 and Cdk4 gene expression, proteins involved in cell division [103]. Additionally, OCN directly and indirectly via glucagon-like peptide-1 (GLP-1), increases insulin production capacity and secretion upon glucose stimulus [103]. While this is shown in in vitro and animal studies, T1DM patients who are given exogenous insulin should technically gain in increased endogenous OCN synthesis, this would then be expected to stimulate pancreatic beta-cell proliferation and subsequent endogenous insulin secretion capabilities. However, T1DM patients do not appear to gain in the upregulation of endogenous insulin production. This may be due to a lack of pancreatic precursor beta-cells, although it has been shown that both T1DM and late-stage T2DM patients do have some functioning precursor pancreatic beta-cells [100,104].

Wei et al. investigated the potential role of OCN as a means to stimulate pancreatic beta-cell proliferation, given T1DM patients retain a small residual population of functional beta-cells [104,105,106], the logic of their hypothesis seems plausible. T2DM patients have low OCN levels too. However, this is with high insulin levels in non-insulin dependent diabetes mellites (NIDDM). The question remains then, would these patients benefit from exogenous OCN therapy? Or similar to conditions of T2DM, patients given exogenous insulin, serves only to mask the downstream problem (hyperglycaemia), while increasing hyperinsulinaemia and insulin resistance, driving the disease further [93,107]. If OCN increases insulin secretion, then would we not see higher levels of OCN in hyperinsulinaemia pathologies such as T2DM, CVD and MetS? On the contrary, those with normal insulin levels have significantly higher OCN levels [93,107]. This may indicate OCN levels and the carboxylation ratio, are firstly markers and then contributory makers of bone fracture resistance, and metabolic and endocrine health.

Using a mouse model, Ferron et al. showed that intermittent injections of OCN improved glucose metabolism, and increased skeletal mitochondrial content which led to improved glucose and fatty acid oxidation capacity. Elevated OCN levels in the absence of hyperinsulinaemia also increased energy expenditure, corroborating other research showing OCN signalling via increasing adiponectin production, leads to increases in brown fat UCP1, resulting in increased thermogenesis. As fat and glucose is oxidised more efficiently, independent of insulin mediated glucose uptake, metabolic markers consequently improve [108].

Evidence suggests the metabolic phenotype of low insulin and glucose levels facilitates maintaining healthy osteoblasts and osteocytes, dendritic connectivity and maintenance of the lacunae-canalicular network. This leads to the metabolic healthy phenotype of higher levels of OCN synthesis and carboxylation capacity, resulting in fracture-resistant bone, with osteocytes maintaining basal bone remodelling. Furthermore, the resultant ability to endogenously produce OCN, enables bone to participate in its endocrine-action on other tissues and organs, further contributing to improved glucose homeostasis and insulin sensitivity [109]. Restriction of dietary carbohydrate intake simultaneously maintains low glucose levels and minimises additional exogenous stimulus on insulin secretion, thus maintaining both markers in the low healthy physiological range.

## 15. cOCN Levels Determine Hydroxyapatite Alignment Formation

Moriishi et al. in *PLOS Genetics*, demonstrated using mouse model OCN knockouts of the two mouse genes for OCN: *Bglap* and *Bglap2*, unlike humans who poses only one OCN gene, that bone apatite crystallite alignment is dependent on carboxylated OCN [110]. In the OCN-deficient mice, bone strength decreased, supporting the classical biology phrase—structure dictates function. In this case, bone strength is determined by its structural quality, which includes bone mass and quality of crystallite alignment with collagen fibres. These structural features, combined, determine bone fracture resistance or osteofragilitas [111]. It is important to note that Moriishi was not investigating OCN deficiency in the context of hyperinsulinaemia, which likely would further contribute other detrimental factors, such as poorer collagen synthesis and increased collagen glycation damage.

Hyperinsulinaemia and hyperglycaemia propagation of impaired osteoblastogenesis and osteocytogenesis results in decreased OCN production capacity, which impairs hydroxyapatite crystallographic orientation to collagen fibrils. Combined with poorer quality collagen synthesis and increased glycated collagen, the summative results may lead to compounding effects on bone fragility via compromised structural quality, that is independent of BMD. The sum of all of these dysregulated/impaired conditions, likely leads to the increased fracture rates seen in patients with the T2DM hyperinsulinaemia-osteofragilitas phenotype.

## 16. The Acute Stress Response

Responses to acute stress increases the ucOCN:cOCN (Glu:Gla) OCN ratio, this decreases OCN bone affinity, which reduces the well-formed structural alignment of hydroxyapatite that is needed for bone fracture resistance [110]. Elevated glucocorticoids increase the synthesis of inflammatory cytokines and tumour necrosis factor α (TNFα). Acute stress signals induce glutamate release from neurites, which competitively inhibits GGCX, resulting in decreased post-translational modification-carboxylation of OCN in osteoblastic lineage cells before cellular release [112,113]. The glutamate concentrations that were shown to achieve this, were on par to that found in glutaminergic synapses [113,114], which were able to cause osteoblasts to increases their release of ucOCN (Glu). This downregulates parasympathetic tone and consequently allows sympathetic signals to be propagated in the absence of suppression. Berger et al. demonstrated that intravenously injected Glu-OCN into WT mice, caused an immediate and significant downregulation of parasympathetic nervous system activity. This causes decreased contraction of the trachea rings via GPRC6A receptor and acetylcholine signalling, with decreased gastrin levels and increased heart rate [113].

Increased ucOCN (Glu) released due to the acute stress response (ASR), leads to inhibition of parasympathetic tone via reducing acetylcholine (ACH) synthesis, release and recycling [113]. Viewed from an evolutionary standpoint, in the event of potential physical trauma, a high stress situation, which may result in a wound, in the insulin sensitive state, the increased ASR inducing ucOCN (Glu) release may be evolutionarily advantageous. ASR ucOCN (Glu) release may induce acute hyperinsulinaemia in-order to effect multiple actions such as facilitating rapid skeletal muscle glucose uptake and aid to inhibit anti-coagulation activity via elevating plasminogen activator inhibitor type 1 (PAI-1) thus disturbing fibrinolysis [53,115,116].

Acute hyperglycaemia provides extra glucose for skeletal muscle, and also increases hepatic clotting factors and clotting activation [115], thus aiding in prevention of excessive blood-loss/haemorrhage from any potential wound and concomitant rapid hypotension due to hypovolemia. Furthermore, elevated blood glucose due to glucocorticoid induced hepatic glycogenolysis and gluconeogenesis, independently stimulates insulin secretion. The ASR induces: acute hyperglycaemia that increases coagulation capacity, bone release of ucOCN (Glu) from osteoblast lineage cells and osteoclastic resorption, together with hyperglycaemia and ucOCN (Glu) induced acute hyperinsulinaemia, which facilitates inhibition of the breakdown of clots. The plausible evolutionary purpose for this, would be to help enable the stabilisation of wound clotting and thus prevent potentially fatal haemorrhage.

## 17. Osteocalcin Regulation of Ketosis

Fasting increases ketogenesis and plasma BHB which increases bone resorption ability via lowering the localised pH level as well as inhibiting/regulating osteoblast mineralisation activity [78]. This would increase plasma OCN levels that would go on to stimulate pancreatic beta cell proliferation and insulin production capacity. Natural diurnal cortisol signalling leads to hepatic glycogenolysis and release of glucose to the system, in turn stimulating insulin secretion. Together, through BHB effect on OCN release, they provide the stimulus for insulin secretion to act as a feedback mechanism to regulate ketogenesis.

OCN is considered to protect against obesity, improve glucose uptake and enhance insulin sensitivity, either directly or via OCN stimulated adiponectin secretion [97,100,103,117,118,119]. Osteocytes comprise the largest population of bone cells, making them likely the biggest producer of OCN than is currently understood. Logically, a substantial loss of osteocytes would result in decreased OCN production and lead to subsequent increases in adiposity. This phenotype is seen in HI/T2DM/CVD patients, who have lower plasma OCN and adiponectin, with increased or normal BMD. Bone mineralisation is possible without OCN and may be enhanced in the hyperinsulinaemic state [110]. Micropetrosis/living-fossilisation of the osteocytic lacuna-canalicular has been demonstrated to be increased in hyperinsulinaemia pathologies [22,23,25,28]. BHB mediated inhibition of mineralisation may be analogous to ePPi and bisphosphonates that help osteocytes maintain their lacunocanalicular network. Inhibition of lacunocanalicular mineralisation concurrent with maintaining osteocyte viability, results in correctly-formed fracture resistant bone that is also dynamically remodelled, a function of healthy bone metabolism leading to wider effects on whole body metabolism.

## 18. Osteocalcin and Insulin Resistance/Hyperinsulinaemia

In vitro and in vivo KO studies, and studies administering exogenous OCN either orally or intravenously to mice or rats, present results that suggest unOCN (Glu) is necessary for pancreatic beta-cell proliferation. OCN is recognised and activates the GPRC6A receptor on pancreatic beta cells [93,120]. This receptor has other ligands; therefore, caution should be applied in interpreting results from genetic GPRC6A receptor KO studies. For example, with *Gprc6a^-/-^* mouse pancreatic beta-cells, knocking out the receptor may have potentially also ablated the role of other ligands in activating this promiscuous receptor and its subsequent downstream intracellular signal cascades. Consequently, there is a possibility of producing different combinatory cell signal results based on ligand type [121]. Furthermore, OCN crosses the blood-brain barrier, and studies have shown a brain target receptor of OCN is *Gpr158*. However, it is likely that this is not the only brain OCN receptor, as studies have shown areas of brain that do not express *Gpr158* whilst still having OCN activity, indicating other receptors at play [100].

In a mouse study, KO of beta-cell *Gprc6a^-/-^* gene expression which encodes the GPRC6A protein receptor, rendered the beta-cells unable to produce sufficient insulin. As a result, these mice had metabolic abnormalities with a similar phenotype to *Ocn^-/-^* KO mice [98,104,120]. However, as the GPRC6A receptor is able to be activated by other ligands, absence of the receptor does not necessarily mean that a lack of ucOCN (Glu) signalling induces the metabolic abnormality. It may be due to the KO inadvertently rendering other ligands to be unable to signal the beta-cells via the “universal multiligand” GPRC6A receptor [121]. An example of the conflicting information from these in vitro and in vivo model studies is found in humans with hyperinsulinaemia pathologies. In these conditions, studies have shown there is a significant inverse correlation between serum OCN levels and: fasting insulin and glucose, BMI, HOMA-IR, leptin and triglycerides (*p* < 0.001), while higher OCN levels positively correlates with adiponectin levels (*p* < 0.001) [93,98]. These real-world investigations appear to contradict the animal model studies, which expect lower OCN levels to predict lower insulin secretion.

Osteocalcin exerts a large amount of its effects via adiponectin [98]. However, under high insulin conditions, adiponectin receptors are downregulated via insulin activating the PI3K/FOXO1 signal transduction pathway. This diminishes the osteocalcin/adiponectin induction of 5’ adenosine monophosphate-activated protein kinase (AMPK), PGC-1α, mitochondrial biogenesis and increased thermogenesis, that all act to facilitate glucose uptake and oxidation independent of insulin [102].

## 19. Vitamin D and Magnesium

Vitamin D is required for osteocyte viability and dendrite connectivity [44]. Hyperinsulinaemia reduces vitamin D availability by increasing sequestration of the lipophilic hormone into adipocytes [122]. Patients with pathologies of hyperinsulinaemia, including T2DM, CVD, obesity, MetS and metabolic cancers, are associated with having a lower vitamin D status [95,123]. In addition, hyperinsulinaemia promotes magnesium deficiency (MgD) which decreases vitamin D transport in the blood [84]. Hyperinsulinaemia decreases hydroxylation of inactive 25-OHD_3_-cacidiol to active 1,25(OH)_2_D_3_-calcitriol by lowering 1-alpha hydroxylase (CYP27B1) activity. Hyperinsulinaemia increases mitochondrial (mt) ROS generation and NAD+ depletion, both decrease NADPH availability [20,56,57]. CYP27B1 activity is NADPH and Mg dependent [84,85,124]. Thus, hyperinsulinaemia decreases cellular capacity to activate vitamin D via lowering CYP27B1 hydroxylase activity.

Chronic hyperinsulinaemia dysregulated vitamin D metabolism negatively affects osteocyte health and their subsequent ability to perform dynamic perilacunar remodelling [44]. A study investigating 783 young northern European males ages 20 to 29 years, found a significant association in vitamin D deficiency with peak bone mass. In participants that had inadequate vitamin D status, parathyroid hormone (PTH) levels and bone-specific alkaline phosphatase (BAP) were higher [125]. Interestingly, a reduction in pre-osteoblast beta-oxidation capacity leads to decreased parathyroid hormone (PTH) sensitivity [126], a potential contributor to the elevated levels of PTH seen in HI/T2DM/CVD patients [127,128].

With the increasing prevalence of pre/diabetes, overweight and obesity in children and adolescents, there are serious implications in long-term health. The negative effect of low vitamin D levels on osteocyte viability consequently negatively affects the OCN-producing cell population; this results in wider implications in bone fracture resistance and metabolic/endocrine health. Vitamin D insufficiency is common in the paediatric population, and is shown to be both a marker and/or maker in increasing the risk of developing pre-diabetes (phenotype 3, stage 1/2, [71]). Early lifestyle interventions that improve vitamin D status, starting from childhood, may contribute to reducing rates of low-energy fractures, and to improving longer-term metabolic health [129].

Insulin is required for healthy bone mineralisation, as seen in insulin insufficient T1DM [130]. Puberty and pregnancy are two stages of development where a natural state of hyperinsulinaemia occurs [77,131,132]. Hyperinsulinaemia enables increased growth in bones during puberty, and mineral accretion during pregnancy, which may be in the form of micropetrosis (osteocyte-lacunae mineralisation), to ensure adequate provision of minerals for nursing offspring during lactation. These phases of life lend a physiological adaptive explanation as to why we would see an increase in BMD in T2DM hyperinsulinaemia [30,32,133]. However, puberty and pregnancy are for a limited duration of time and come with either growth in bones (puberty), or lactation and subsequent resorption of bone minerals. Whereas T2DM hyperinsulinaemia may go undetected for many years, a pernicious chronic elevation, leading to excessive micropetrosis and living fossilisation. The resultant disconnecting of osteocytes from one another, impairs their ability to: sense and transmit information, modulate one another, and directly/indirectly modulate bone turnover [31,62,134]. This hypothesis provides a plausible explanation as to why we would see normal to H-BMD in T2DM not conferring fracture resistance.

Rolvien et al., found a 43% greater decrease in empty lacunae fraction with a decrease in the number of viable osteocytes in vitamin D deficient versus replete human iliac crest biopsies (*p* < 0.001) [44]. Given that hyperinsulinaemia decreases vitamin D availability, activation and transport, this is likely to profoundly influence osteocyte directed bone remodelling, as osteocyte viability and thus population is vitamin D dependent. Interestingly, osteocytes express the CYP27B1 enzyme, and are able to directly activate 25(OH)D_3_-calcidiol to 1,25(OH)_2_D_3_-calcitriol, providing a localised refined control in provision of active vitamin D availability. One of calcitriol’s many roles is to regulate/suppress nuclear factor-kB (NFkB) pathway signalling in the adaptive immune system [135,136]. Theoretically, given osteocytes modulate osteoclastogenesis from the myelopoietic monocyte/macrophage cell lineage, it stands to reason that their ability to directly activate vitamin D serves to locally modulate/fine tune NFkB signalling, which would function in the regulation of osteoclastogenesis. However, this needs to be investigated further.

## 20. Hyperinsulinaemia Decreases Vitamin D Availability, Decreasing Osteocalcin Synthesis

Bioactive 1,25(OH)_2_D_3_-calcitriol stimulates OCN transcription. Vitamin D response elements are found within the osteoblast/osteocyte 600-nucleotide OCN gene regulatory-sequence transcription start site [137]. Hyperinsulinaemia increases de novo lipogenesis, and drives lipophilic vitamin D to be accreted into adipocytes [122], decreasing availability. Vitamin D deficiency favours osteocyte apoptosis and reduced osteocyte connectivity, decreasing their viability further. Osteocyte connectivity is essential for their function in healthy bone remodelling, and to effect regulation of OCN production [44,135,138,139].

The osteocytes expression of CYP27B1, enabling localised direct activation of calcidiol to calcitriol [41,136], arguably facilitates osteocyte regulation of osteoblastic OCN production. Increased presence of differentiated osteocytes positively correlates with gene expression of OCN in osteoblasts, indicating osteocyte control of osteoblast OCN synthesis [140]. If osteocyte population/health is compromised, this may in turn compromise osteoblast OCN production. A cross-sectional study of 191 non-osteoporotic postmenopausal women found a significant negative correlation between serum OCN and insulin resistance (IR) and HbA1c, *p* = 0.001 and *p* = 0.048 respectively [95]. Hyperinsulinaemia patients have lower serum OCN levels than healthy persons, supporting this notion. However further investigations are needed.

## 21. Vitamin K, Osteocalcin Carboxylation and Hydroxyapatite Crystallite Alignment

Hyperinsulinaemia mediates de novo lipogenesis and adipogenesis, inhibits lipolysis, and drives adipocyte vitamin K sequestration [95]. This leads to decreased vitamin K availability for OCN carboxylation. Vitamin K_1_ sequestration into adipocytes is found in higher concentrations in adipose tissue. The reduced form of vitamin K_1_ is an essential co-factor for the γ-glutamyl carboxylase enzyme, to carboxylate OCN [112]. Children who have suffered low energy fractures versus never-suffered-fractures controls, have significantly lower levels of serum carboxylated OCN to uncarboxylated (cOCN:ucOCN) [117]. This corroborates OCN requirement for hydroxyapatite crystallite alignment with collagen for bone quality, which includes the mineralisation structural formation as well as BMD, which translates into fracture resistance [99,110].

Lower levels of vitamin K has also been found to be negatively correlated with ucOCN (Glu) levels in healthy women, where elevated ucOCN (Glu) is a known risk factor for increased fracture risk [141]. In a double blinded, randomised controlled trial with T2D patients, *n* = 40, between 30 to 70 years of age, patients were assigned to one of three groups: (a) placebo-a, *n* = 16, 1000 IU vitamin D3 + calcinated magnesium, (b) placebo-b, *n* = 12,100 ug vitamin K2 + calcinated magnesium and (c) the intervention group, *n* = 12, 1000 IU vitamin D3 + 100 ug vitamin K2. The vitamin D3 group had a significant decrease in serum ucOCN (Glu) 3.3 ± 1.7 ng/dL to 2.5 ± 1.5 ng/dL (*p* = 0.026), ucOCN:cOCN (Glu:Gla) ratio 7.0 ± 7.0 to 3.1 ± 1.7 (*p* = 0.039), glucose (*p* < 0.001) and pancreatic beta cell percentage function (*p* = 0.041). The vitamin K2 group saw a significant decrease in glycaemia (*p* = 0.002), pancreatic beta cell functional percentage (*p* = 0.039), HOMA-IR (*p* = 0.041) and a significant increase in cOCN (Gla) from 0.818 ± 0.567 ng/dL to 1.2 ± 1.1 ng/dL (*p* = 0.041). The group receiving both vitamin D3 and K2, saw a significant decrease in glycaemia (*p* = 0.002) with a decrease in pancreatic beta cell function (*p* = 0.004), and a significant decrease in ucOCN:cOCN (Glu:Gla) from 6.4 ± 4.2 to 4.3 ± 2.9 ratio (*p* = 0.023) [101]. An interesting aspect of this is the decrease in glycaemia in the context of a decrease in functional pancreatic beta cells, which seems counter-intuitive. This indicates decreased reliance on insulin for glucose homeostasis, which supports other studies that show both cOCN and ucOCN are biologically active and have a functional role in insulin-independent glucose uptake. In addition to increasing adipocyte and myocyte mitochondrial biogenesis and uncoupling proteins, leading to increased thermogenesis, likely via stimulating adiponectin synthesis [98].

## 22. Glycation Damage Decreases Vitamin K-Dependent Carboxylation of Osteocalcin

As described earlier, cOCN is required for healthy bone matrix hydroxyapatite crystallite alignment, which confers structural integrity to bone that decreases fracture risk. Carboxylation of OCN is mediated by the vitamin K dependent γ-glutamyl carboxylase enzyme [112]. Lipophilic vitamin K is transported via chylomicron remnants (CR) in the plasma [142]. Osteoblasts and osteocytes express the membrane receptor proteins apolipoprotein E (apoE) and LDL receptor related protein 1 (LRP-1). A ligand for osteoblast LRP-1 is the CR apoE protein. Osteoblasts are thus able to take up CR containing vitamin K via LRP-1 recognition and binding with the CR structural apoE protein leading to receptor mediated endocytosis. LRP-1 recognition of apoE is via the heparan sulfate proteoglycan (HSPG) mediated pathway [143,144]. Uptake of CR cargo: vitamin K and dietary lipids, are required for osteoblasts’ high metabolic demand. Hyperinsulinaemia negatively impacts HSPG function and availability, via impairment of vitamin D regulation. Vitamin D regulates sulfate synthesis, required for heparan sulphate [145].

Neimeier et al. demonstrated in a murine in vivo model that osteoblast internalisation of CR, requires osteoblast membrane expression of endogenous apoE in order to tether CR in the first steps of endocytosis uptake. This occurs in a similar secretion-recapture mechanism performed by hepatocytes [142]. ApoE is highly susceptible to sugar moieties irreversibly attaching to NH_2_-protein groups. This glycation damage interferes with CR attraction, receptor recognition and lipid-binding ability [144,146], and ultimately prevents the uptake of CR carrying vitamin K and fatty acids to osteoblasts.

## 23. Hyperinsulinaemia Decreases Heparan Sulphate Proteoglycans

Heparan sulphate proteoglycans are robust anticoagulants, buffering glycation damage. An increase in heparanase and a decrease in heparin sulphate is implicated in endothelial cell dysfunction [147]. Heparanase is an endoglycosidase that enzymatically cleaves glycosaminoglycan heparan sulphate [148]. Hyperglycaemia oxidative damage, resulting in AGE, and receptor for AGE (RAGE) production, increases heparanase expression [147,149]. Heparan sulphate is required for adipocyte to macrophage mitochondrial transfer, which is decreased in hyperinsulinaemia/obesity [150]. Hyperinsulinaemia drives lowered vitamin D hydroxylation/activation/transport, increasing sulphate wastage and oxidation damage to heparin sulphate proteoglycans [145]. The multiple mechanisms by which hyperinsulinaemia and hyperglycaemia decrease extracellular-localised heparan sulphate, contribute to impairing osteocytes and osteoblast uptake of CR cargo, including essential fatty acids for fuelling and vitamin K. These further drive cellular reliance on glucose fuelling, in turn depleting NAD+ and increasing ROS. Perlecan is a form of heparan sulphate proteoglycan that is essential in positioning, anchoring and receiving mechano-stimuli for osteocytes in their lacunocanalicular space. Hyperinsulinaemia and hyperglycaemia driven breakdown of HS via increased heparanase enzymatic cleavage and increased sulphate wastage, results in decreasing osteocyte perlecan, further contributing to harming osteocyte viability and function [31,62].

Thus the “perfect storm” is established in hyperinsulinaemia osteofragilitas, with increased glycation damage to: collagen rendering it rigid and unrecognisable to digestion enzymes, the vasculature causing increased hypoxia, and to apoE proteins that are essential for receptor mediated uptake of vitamin K. Without vitamin K, osteocalcin carboxylation status decreases, resulting in disordered hydroxyapatite crystallite formation and consequent increased bone fragility irrespective of BMD. Furthermore, glycation damage to apoE and HSPG would decrease delivery of fatty acids for the high metabolic demands required for osteoblast collagen synthesis and osteoblasto/cytogenesis. Impaired fatty acid delivery, along with insulin-mediated inhibition of beta-oxidation and increased mtROS generation, consequently drives poorer collagen production and decreased OCN synthesis. With reduced ability to access fatty acids for fuel, concomitant to increased hypoxia, osteoblasts and osteocytes are forced to be more reliant on glucose oxidation, leading to increased mtROS production, which decreases osteocytogenesis and osteocyte dendritic connectivity [59]. Dynamic bone remodelling decreases and a vicious cycle ensues.

## 24. OCN and the Brain

OCN protects against neural apoptosis and enhances hippocampus neurogenesis. OCN has been shown to induce changes in GABA and neurotransmitter gene expression, decreasing GABA and increasing serotonin and dopamine, ameliorating anxiety and depression, and improving learning capacity [151]. Oury et al. showed in mice studies and in ex vivo and explant studies; OCN affects neurotransmitter gene expression in the brain. They cleverly demonstrated that OCN passes through the BBB. OCN mediates these neural effects via a different receptor to GPRC6A, as they conducted the same experiments in *Gprc6a^-/-^* knockout and *Ocn^-/-^* mice. This study also showed administering of OCN rescued the neurological effects in learning. Mice pups born to *Ocn^-/-^* mothers suffered significant increased anxiety, depression and loss of learning capability. Furthermore, neurogenesis was negatively affected. The ability to learn was assessed via the Morris Water Maze test (MWM), which is considered a test that measures the function of the hippocampus. Mice born from *Ocn^-/-^*, WT, *Esp^-/-^* mothers were tested 4 times a day for 10 days. The *Ocn^-/-^* mice results showed that, over the 10 days of “exposure to the activity,” they were almost completely unable to learn [151].

Khrimian et al. provides evidence that OCN positively regulates hippocampal-dependent memory by activating the inositol 1,4,5-triphosphate (IP3) intracellular signal transduction cascade via binding to neuronal Gpr158 in the CA3 region of the hippocampus [152]. This area of research is in its infancy and warrants further investigation, given the increase in cognitive decline, Alzheimer’s disease (AD) and Parkinson’s disease (PD) that are increasingly recognised as conditions of hyperinsulinaemia and mitochondrial distress [153,154,155]. Increasing evidence is demonstrating a role of OCN in neurological health and disease [109,156,157]. This leads to further concerns with the increasing earlier rates of obesity, T2D and hyperinsulinaemia in women. Evidence suggests that maternal OCN levels may impact embryonic neurogenesis and rescue from apoptosis, leading to long term effects on offspring such as anxiety, depression and learning capacity [151]. AD and PD are associated with significant increased rates of fragility fractures, furthermore, fragility fractures are also associated with an increased rate in the development of dementia [158,159,160,161].

## 25. Osteocalcin Affects Satiety Regulation and Hepatic Glucose Output

OCN increases serum glucagon-like peptide-1 (GLP-1) levels [162]. GLP-1 suppresses pancreatic α cell secretion of glucagon leading to glycaemia regulation (decreasing hepatic glucose output). GLP-1 also slows gastric emptying leading to increased nutrient absorption, and inhibits food intake [163]. The slowing of gastric emptying and inhibition of food intake by GLP-1 is mediated via vagal circuits, as this effect was shown to be abrogated after truncal vagotomy in normal weight none-diabetic men [164].

OCN further modulates pancreatic α cell secretory profile via regulating gene expression of the rate limiting enzyme tryptophan hydroxylase (*Tph*) for serotonin (5-hydroxytryptamine) synthesis from 5-hydroxytryptophan (5-HTP) [151,154,165,166,167,168]. Under high and low glucose settings, the pancreatic alpha cell glucagon secretion decreases when serotonin levels are higher [71,168]. OCN levels are higher in healthy individuals and lower in people with T2D. This is potentially a contributory factor in lost-inhibition on glucagon secretion, therefore leading to increased hepatic glucose output observed in T2 diabetics. The consequent higher set point of hepatic glucose output, feeds forward in generating higher rates of glycation damage and hyperinsulinaemia.

Interestingly, OCN survives the digestive tract intact and is biologically active in the gut. Mizokami et al. demonstrated in mice, that oral ingestion of OCN was more effective at maintaining serum GLP-1 levels than via intraperitoneal injection. The digestive form likely works without absorption via stimulating GPRC6A on the apical side of the intestinal enterocytes, to stimulate synthesis of GLP-1, without the need to enter into the blood stream [162]. GLP-1 is rapidly degraded by cell-surface aminopeptidase dipeptidyl peptidase IV (DPPIV) [163]. Around 4% of oral OCN is absorbed into the blood stream, and raises serum GLP-1 as much as intravenous injection [162]. It may be that OCN action within the gut, on the apical surface side of epithelial cells works in addition to basal side activation, effectively sustaining synthesis for a longer duration of time, independent of raising serum OCN levels. GLP-1 agonists are currently an exciting area of research in the management of T2DM, aiding in decreasing hepatic glucose output.

## 26. Laboratory Identification

Patients presenting with low energy fracture(s) and normal to higher BMD should be investigated for T2DM and hyperinsulinemia. Furthermore, identification of hyperinsulinaemia-osteofragilitas before fracture occurrence will enable earlier intervention, and provide better patient understanding and compliance with clinical nutritional management. Laboratory identification biomarkers to be evaluated include: fasting insulin, glucose, BHB, OCN (ideally the cOCN:ucOCN ratio to be included), glucagon, GLP-1 and serotonin. If BHB levels are below 0.1 mmol/L and OCN levels are on the lower end or below the reference range, a 5-h OGTT with insulin sensitivity assay in-order to determine Kraft pattern and metabolic phenotype is advised [16,71].

An effective method to decrease excess insulin exposure whilst simultaneously improving glucose homeostasis, for hyperinsulinaemia with or without hyperglycaemia, is through lifestyle management. Carbohydrate restriction with adequate individualised support, should be considered the first line of treatment [53,169,170], with further focus on a diet that maximises nutrient density, especially: vitamin D3, K and magnesium.

## 27. Conclusions

Micropetrosis/living fossilisation increases with age. Further investigations need to be conducted into the presence and degrees of micropetrosis in hyperinsulinaemia-osteofragilitas. Furthermore, hyperglycaemia decreases osteoclastogenesis [7], which would inhibit the bone resorption that is necessary to effect bone remodelling, which would include the need for removal of glycated collagen and micropetrotic bone. However glycated collagen is rendered unrecognisable by enzymes for breakdown [47]. In addition to increased glycation of HbA1c and collagen in hyperinsulinaemia T2DM, MetS and CVD patients [146], there is also increased glycation of their ApoE and ApoB lipoproteins [146]. This renders chylomicron remnants carrying vitamin K, unable to be recognised by LRP-1 receptors on osteoblast and osteocytes [142]. Carboxylation capacity will be decreased as a result of reduced vitamin K dependent γ-glutamyl carboxylase activity, along with increased bone fragility and fracture risk due to poorer structural alignment of bone hydroxyapatite, which is dependent on carboxylated OCN status [110]. Osteocytes synthesize OCN and directly control osteoblast OCN synthesis. Osteocyte dendritic connectivity and viability, and their capacity to maintain the lacunocanalicular fluid-filled network is evidently essential, in order for them to perform their role in orchestrating and regulating dynamic bone remodelling. This is not only a marker and maker of healthy bone, but is also increasingly appearing as a key endocrine player in whole body metabolism, as well as many homeostatic feedback loops.

Pregnancy and seasonal feasting (autumn where fruit would be more abundant) are two natural events are recognised to induce a period of physiological hyperinsulinaemia [77], where it is plausible to reason that the hyperinsulinaemia phenotype would not only enable the increase in adipogenesis for energy storage, but also facilitate vitamin and mineral storage. Hyperinsulinaemia drives increased adipocyte sequestration of vitamin D and K. Vitamin D is required for osteocyte viability and dendritic health, and to regulate OCN synthesis. Healthy osteocytes synthesise OCN and stimulate osteoblast OCN synthesis. The carboxylation status of OCN is dependent on vitamin K availability. Hyperinsulinaemia decreases vitamin K circulation availability, whilst the increased glycation to proteins occurring in hyperinsulinaemic and hyperglycaemic patients renders vitamin K delivery and uptake via CR apoE/LRP-1 receptor-mediated uptake seriously impaired. Consequently, less OCN is synthesised and less is carboxylated, resulting in poorly formed bone hydroxyapatite crystallite alignment, yet not necessarily compromising BMD, resulting in hyperinsulinaemia-osteofragilitas. Furthermore, glycation damage is incurred on existing bone type 1 collagen, rendering it stiffer and unrecognisable to digestive enzymes. Glycation-induced inhibition of CR fatty acid delivery to osteoblasts and osteocytes results in decreased beta-oxidation, which is needed for the high energy demand for collagen synthesis—at the same time, increasing mtROS production due to an increased reliance on glucose-derived ATP synthesis, which depletes intracellular NAD+. This decreases mitochondrial antioxidant enzyme synthesis, culminating in the production of poorer quality collagen.

Both seasonal diet-induced hyperinsulinaemia and the time limited duration of pregnancy hyperinsulinaemia increase osteocyte spherite-mediated living fossilisation, thus entombing the osteocytes along with magnesium and other minerals within bone, increasing BMD in the process. In a healthy natural cycle, winter follows autumn and lactation follows pregnancy, where minerals would need to be released back into the system. However, chronic hyperinsulinaemia is a situation where winter never comes. The continual stimulant of insulin secretion largely due to dietarily derived farinaceous carbohydrates, perpetuates the physiological condition into a pathological condition as threshold limits are passed, such as the degree in loss of osteocytes and degree of living fossilisation of their lacunocanalicular system, that may no longer be easily recoverable. The consequences are further reaching than simply bone fracture resistance, as we are increasingly discovering bone’s role in cardiovascular, muscular, renal and potentially neurological health. Keeping bone healthy has much wider implications in chronic diseases and the ageing process. Evidence suggests that carbohydrate restriction that maintains a healthy low insulin and glucose level, also positively maintains the viability of osteocytes and osteoblasts, that orchestrate dynamic bone remodelling, bone strength and fracture resilience. In turn, maintaining healthy osteocytes, likely feeds forward in positively modulating neurological, endocrine and metabolic health.
